# The Impact of Rheological Characteristics on the Swallowing Dynamics of Xanthan Gum-Based Thickeners

**DOI:** 10.3390/foods15030461

**Published:** 2026-01-28

**Authors:** Yuki Hayakawa, Jumpei Okawa, Yuzuki Izumi, Rikako Sato, Satomi Kawakami, Hirofumi Sonoki, Kazuhiro Miyaji, Takahiro Ono, Kazuhiro Hori

**Affiliations:** 1 Division of Comprehensive Prosthodontics, Graduate School of Medical and Dental Sciences, Faculty of Dentistry, Niigata University, Niigata 951-8514, Japan; y-hayakawa@morinagamilk.co.jp (Y.H.); jookawa@dent.niigata-u.ac.jp (J.O.); rikako310@dent.niigata-u.ac.jp (R.S.); 2 Health Care and Nutritional Science Institute, Morinaga Milk Industry Co., Ltd., Zama 252-8583, Japan; yuzuki-izumi412@morinagamilk.co.jp (Y.I.); s_kawakm@morinagamilk.co.jp (S.K.); h-sonoki@morinagamilk.co.jp (H.S.); k_miyazi@morinagamilk.co.jp (K.M.); 3 Department of Gerodontology, Osaka Dental College, Osaka 540-0008, Japan; ono-t@cc.osaka-dent.ac.jp

**Keywords:** dysphagia, thickener, xanthan gum, swallowing dynamics, rheological properties

## Abstract

Xanthan gum-based thickeners are commonly used to treat dysphagic patients. Their rheological properties, such as shear-thinning, extension, and thixotropy, contribute to swallowing safety. However, the influence of rheological variations on swallowing dynamics remains unclear. This study investigated the impact of variations in the rheological properties of xanthan gum-based thickeners on swallowing dynamics, specifically tongue pressure, suprahyoid muscle activity, and swallowing sounds. Shear rheology, extensional viscosity, and 3-interval thixotropy tests were conducted on three commercial thickener solutions standardized to a 400 mPa·s viscosity at a 50 s^−1^ shear rate. Twenty healthy volunteers (11 females, 24.6 ± 2.4 years) participated in this study, during which tongue pressure, suprahyoid muscle activity, and swallowing sounds were measured while swallowing 15 mL samples. The first thickener exhibited reduced shear viscosity at 300 s^−1^, higher thixotropy, and shorter swallowing sound duration, suggesting a shortened pharyngeal transit time. The second showed prolonged filament breakage time and higher tongue pressure in the posterior-median region of the palate, leading to increased tongue activity during swallowing. The third exhibited lower extensional viscosity, different muscle activity than the second, and longer duration of swallowing sound than the first. These results suggest that rheological property variations in xanthan gum-based thickeners influence swallowing dynamics in healthy individuals.

## 1. Introduction

For dysphagic patients, who have difficulty in swallowing, texture-modification diets (TMDs) rely on thickeners to increase the viscosity of liquids, thereby decreasing bolus flow through the pharynx and reducing the risk of aspiration and laryngeal penetration [1]. Thickeners can be categorized into two groups: modified starch-based and gum-based thickeners. Xanthan and guar gums are commonly used gum-based thickeners that increase viscosity. Gum-based thickeners have gained popularity owing to their favorable sensory attributes, such as smooth texture, taste, and appearance [2,3,4]. Among them, xanthan gum-based thickeners are widely utilized in many products owing to their high clinical safety and efficacy [5]. Our previous study comparing post-swallow pharyngeal residues in starch, guar gum, and xanthan gum-based thickeners found that xanthan gum-based thickeners were the most clinically effective, as evidenced by the lower prevalence of pharyngeal residues [6]. This finding is consistent with the widespread adoption of xanthan gum-based thickeners in clinical practice.

The optimal viscosity levels of thickened fluids prescribed to individual patients vary depending on the severity of dysphagia. Various countries, including the USA, Australia, Canada, and Japan, classify thickened fluids based on their viscosity at a shear rate of 50 s^−1^ [7]. However, differences in sensory properties, such as stickiness, have been observed among xanthan gum-based thickeners adjusted to the same viscosity at a shear rate of 50 s^−1^ [8]. Furthermore, a clinical study [9] evaluating the optimal viscosity level at a shear rate of 50 s^−1^ using xanthan gum-based thickeners suggested that the unique rheological properties of the product may contribute to the effectiveness of safe swallowing.

Thickened liquids typically exhibit non-Newtonian behavior. An important rheological property of thickened liquids is shear-thinning, which refers to a decrease in viscosity with increasing shear rate. A report evaluating the shear-thinning effect of commercially available thickeners [10] revealed that all samples demonstrated shear-thinning, with varying flow indices among products. The shear rate during pharyngeal transit is estimated to be 300 s^−1^, and the shear-thinning property during pharyngeal transit is believed to be related to swallowing safety. Additionally, the extensional properties of thickened liquids are thought to be related to swallowing dynamics. As the bolus moves from the oral cavity to the pharynx during swallowing, shear and extensional deformations occur simultaneously. Hadde and Chen [11] demonstrated that extensional deformation is associated with bolus cohesiveness and can help prevent aspiration. Studies comparing commercially available thickeners have demonstrated that at the same shear viscosity, differences in extensional properties are observed among xanthan gum-based thickeners [4]. Recent findings have suggested that the thixotropic properties of thickened liquids, including those containing xanthan gum, may be crucial for safe swallowing [12]. Thixotropy, a unique property of polysaccharides that result in the formation of microstructures in solution, is also present in xanthan gum. The time-dependent breakdown and regeneration of these microstructures during bolus flow could be linked to swallowing safety [13]. However, the relationship between these rheological properties and swallowing dynamics in xanthan gum-based thickened liquids remains unclear.

This lack of clarity stems from the fact that rheological properties reflect mechanical responses to material deformation and flow. It is therefore challenging to accurately assess these forces using conventional clinical methods, such as videofluoroscopic and endoscopic observations. We developed a tongue pressure sensor sheet that measures the force generated between the tongue and palate in real time as the bolus is propelled into the pharynx [14]. Our previous study demonstrated that tongue pressure measurement reveals a strong temporal coordination between tongue pressure generation and hyoid movement during swallowing, establishing tongue pressure as a valuable quantitative indicator of swallowing function [15]. This method can be used to compare bolus propulsion forces during oral transport when consuming boluses thickened with different xanthan gum-based thickeners. In addition, by evaluating swallowing sounds during the pharyngeal phase, which corresponds to pharyngeal transit time [16], and suprahyoid muscle activity during swallowing, the relationship between rheological properties and swallowing dynamics can be clarified. Our hypothesis is that variations in the rheological properties of xanthan gum-based thickeners may impact swallowing dynamics. The objective of this study was to elucidate the distinctions in shear-thinning, extensional properties, and thixotropy among three xanthan gum-based thickening products. Additionally, we aimed to investigate the effects of these differences in properties on tongue pressure, swallowing sounds, and suprahyoid muscle activity during swallowing in healthy individuals.

## 2. Materials and Methods

### 2.1. Materials and Preparation Methods

Three commercial xanthan gum-based food thickeners for managing dysphagia were selected for this study: Tsururinko Quickly (TQ; Morinaga Milk Industry Co., Ltd., Tokyo, Japan), Toromi Smile (TS; Healthy Food Co., Ltd., Tokyo, Japan), and Resource Thicken Up Clear (RC; Nestle S.A., Barcelona, Spain). To ensure precise injection of the sample into the oral vestibule during physiological measurements, an edible green dye (Melon Color B, San-Ei Gen F.F.I., Inc., Toyonaka, Japan) was added to distilled water at 0.01 *w*/*v*%, making it visually observable. The colored distilled water (300 g) was placed in a 500 mL beaker, and while stirring at a speed of four rotations per second using a 180 mm long spatula, TQ (10.2 g), TS (8.9 g), or RC (15.0 g) was added and stirred for 40 s to achieve a steady shear viscosity of 400 mPa·s at a shear rate of 50 s^−1^. The amount of thickener was determined based on the viscosity value measured 2 min after the initiation of the measurement, at which point the viscosity had stabilized [8]. Subsequently, the mixture was left to stand at room temperature (20 °C) for 1 h and then utilized for the rheological and physiological measurements.

### 2.2. Shear and Extensional Rheology

Shear rheological measurements were conducted using a stress-controlled rheometer (MCR301, Anton Paar, Graz, Austria) equipped with a cone-and-plate viscometer (50 mm in diameter, 1° cone angle, and 0.1 mm gap). The shear-thinning effect was assessed over a shear rate range of 0.01–1000 s^−1^ at 20 °C, with each measurement performed three times for each sample. Viscosity values at shear rates of 40 s^−1^ and 251 s^−1^ were compared among the three samples. In addition, the obtained values were fitted using the model power-law equation described by Hadde and Chen [11]. This equation, η = K$\dot{\gamma }$*n*^−1^, was fitted to the data for shear viscosity as a function of shear rate to calculate the flow consistency index (K) and flow index (*n*). The values of K and *n* obtained from the fitting results were then compared among the three samples.

The extensional viscosity and filament breakage time were measured using a method described in a previous study [17]. Surface tension was measured at 25 °C on a Wilhelmy-type instrument DY-500 (Kyowa Interface Science Co., Ltd., Saitama, Japan). Extensional viscosity was determined using the surface tension and filament diameter at 25 °C with a capillary breakup extensional viscometer (CaBER1, Thermo Haake GmbH, Karlsruhe, Germany). All measurements were performed three times, and the filament breakage time, maximum extensional viscosity, and extensional viscosity at each Hencky strain were compared across the three samples.

Thixotropy was assessed through the three-interval thixotropy test (3ITT) measurement method developed by Toker et al. [18]. The temporal behavior of structural recovery for each sample was evaluated by measuring the storage modulus G′ in a test comprising the following three intervals:

First interval: resting at a frequency of 1 Hz and strain amplitude of 5% within the linear viscoelastic region (0–120 s).

Second interval: structural deformation at a high shear rate of 300 s^−1^ (121–240 s).

Third interval: structural recovery at a frequency of 1 Hz and strain amplitude of 5% (241–540 s).

The measurement was conducted three times for each sample at 20 °C. The storage modulus G′ in the first and third intervals was compared across the three samples. Additionally, in this test, the recovery rate (%Recovery) after structural deformation was calculated from the value of G′ in the third interval relative to the value of G′ at 120 s in the first interval. Furthermore, the recovery rate was fitted using the following equation, referring to a previous study [19]:

%Recovery = α_1_ (1 − exp(−k_1_t)) + α_2_ (1 − exp(−k_2_t)) + β

where k_1_ and k_2_ represent the time constants for short and long times, respectively. Based on the results, the coefficients α_1_, α_2_, k_1_, and k_2_ were compared across the three samples.

### 2.3. Evaluation of the Swallowing Dynamics

#### 2.3.1. Participants

Twenty healthy participants (9 males and 11 females; 24.6 ± 2.4 years old) without abnormalities in the number or position of teeth except for the third molar, abnormality in occlusion, mastication, or swallowing disorder, a history of neuromuscular diseases, and allergy to the test samples were included in this study. All participants were fully informed of the study purpose, and written consent was obtained. This study was conducted in accordance with the Declaration of Helsinki and approved by the Ethics Review Committee of Niigata University (2019-0294).

#### 2.3.2. Equipment

Muscle activity, tongue pressure, and swallowing sounds were investigated simultaneously. The aforementioned parameters were synchronized by inputting the synchronization signal from the tongue pressure sensor sheet system onto an A/D board (Power Lab ML880, AD Instruments, Bella Vista, Australia).

##### Muscle Activity

The activity of the suprahyoid muscles during swallowing was assessed using a wireless electromyograph (Trigno Wireless EMG System; Delsys Inc., Natick, MA, USA). The electrodes were attached to the equivalent anterior bellies of the left and right digastric muscles. Data was sampled at 1 kHz and recorded on a PC through an A/D board (Power Lab ML880; AD Instruments, Bella Vista, Australia). The recorded electromyogram signals were full-wave rectified and smoothed over 20 ms.

##### Tongue Pressure

Tongue pressure was measured using a Swallow Scan System (Nitta, Osaka, Japan) in this study [14]. The sensor sheet of this system, T-shaped and approximately 0.1 mm-thick, was placed on the hard palate using a denture stabilizer (Touch Correct II, Shionogi, Tokyo, Japan). It featured pressure-sensitive points at the following five locations crucial for measuring tongue pressure during swallowing: the anterior-median region (Ch.1), mid-median region (Ch.2), posterior-median region (Ch.3), posterior circumferential region on the right (Ch.4), and posterior circumferential region on the left (Ch.5). Data obtained from the measurements were recorded on a personal computer at a sampling rate of 125 Hz.

##### Swallowing Sound

The sound of the bolus passing through the pharynx during swallowing was captured using a microphone (SH-12jk, Nanzu, Shizuoka, Japan). The microphone was positioned to capture sound beneath the edge of the cricoid cartilage [20,21]. The sampling rate was set at 40 kHz, and the data were recorded on a personal computer using an A/D board (Power Lab ML880; AD Instruments, Bella Vista, Australia).

#### 2.3.3. Data Collection

Participants were instructed to sit in the chair and maintain an upright posture. Their heads were supported by a headrest to prevent head movement and ensure that the Frankfort plane remained horizontal; they were instructed to keep their feet firmly planted on the floor. The researcher injected the samples into the oral vestibule using a syringe, instructing the participants to swallow the sample in one swift motion [22]. The samples comprised thin liquid and liquids thickened by TQ, TS, and RC, with each participant receiving 15 mL of sample at room temperature for measurement. All samples were consumed three times at random.

#### 2.3.4. Data Analysis

**Figure 1:**
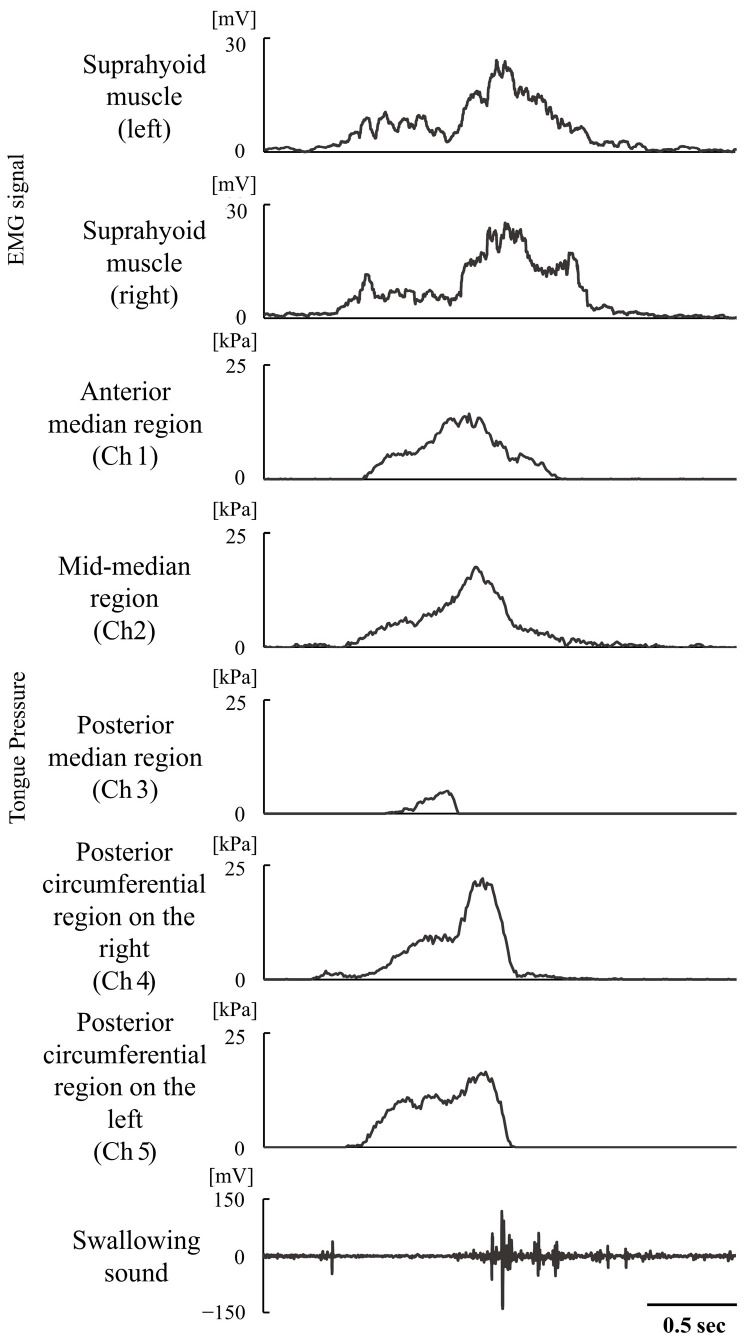
Muscle activity, tongue pressure, and swallowing sound.

EMG signals of suprahyoid muscle activity, tongue pressure, and swallowing sound were recorded and are shown representatively in Figure 1. The maximum amplitude, duration, and integral value of suprahyoid muscle activity were calculated from the electromyography waveforms on both sides. Next, the maximum value, duration, and integral value of tongue pressure in Ch.1–5 were calculated from the tongue pressure waveforms. The duration of the swallowing sound, observed after the onset of muscle activity, was also calculated [23]. The onset of muscle activity, tongue pressure, and swallowing sound were identified as the points at which the amplitude exceeded twice the standard deviation of the baseline amplitude of each waveform. Conversely, the offset of these activities was determined as the points at which the amplitude dropped below twice the standard deviation of the baseline amplitude of each waveform. The average values from the three trials for each participant were used as the definitive values for each sample swallow and were used for comparison across samples.

### 2.4. Statistics

To compare the shear-thinning, extensional rheology, and thixotropic properties, multiple comparisons were performed using the Tukey method. Statistical significance was set at *p* < 0.05, which was considered significant. In addition, after confirming normality and equal variance, each result of suprahyoid muscle activity, tongue pressure, and swallowing sound was analyzed using a linear mixed model with the sample as a fixed effect and the subject as a random effect. Multiple comparisons were performed using Tukey’s method for intergroup differences in the sample. All statistical analyses were performed using JMP software version 13 (SAS Institute, Cary, NC, USA).

## 3. Results

### 3.1. Shear and Extensional Rheology

#### 3.1.1. Shear Viscosity

**Figure 2:**
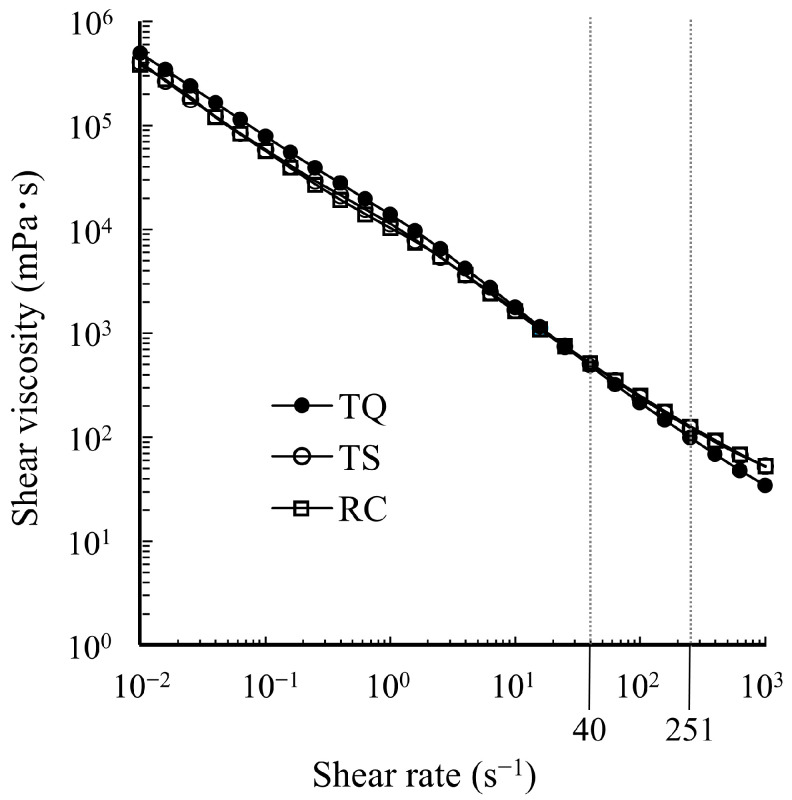
Shear rate dependence of the viscosity of TQ, TS, and RC.

The shear viscosity profiles of the samples are shown in Figure 2, demonstrating a decrease in viscosity as the shear rate increased across all samples. No discrepancy in viscosity was observed at a shear rate of 40 s^−1^ among the samples; however, the viscosity of TQ at a shear rate of 251 s^−1^ was significantly lower than that of TS and RC (Table 1, *p* < 0.05). The fitting results based on the power-law model are listed in Table 1. The flow index (*n*) of TQ was significantly lower than those of TS and RC (*p* < 0.05).

#### 3.1.2. Extensional Viscosity

**Figure 3:**
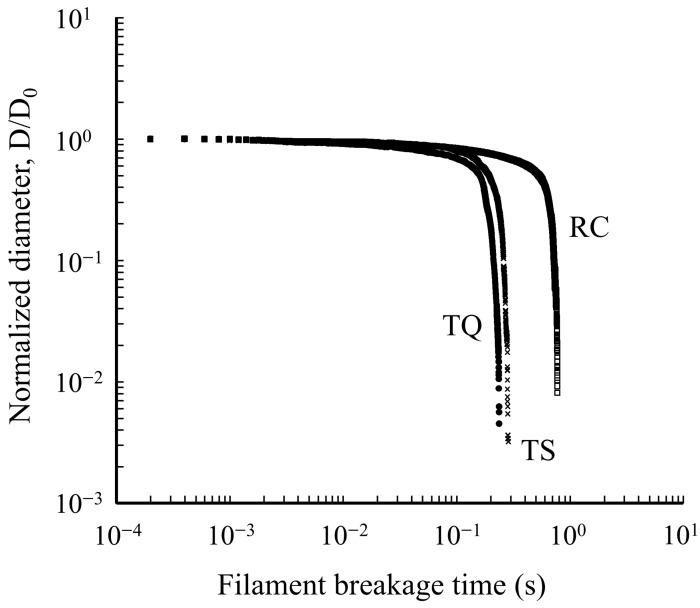
Changes in filament diameter measured at the midpoint and normalized by the initial (D/D_0_) for TQ, TS, and RC.

**Figure 4:**
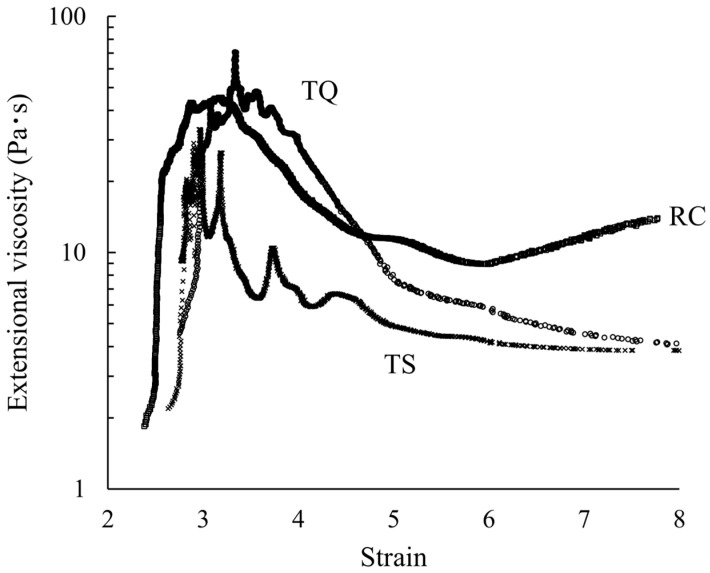
Representative examples of Hencky strain dependence for TQ, TS, and RC.

The relationship between filament diameter and filament breakage time is shown in Figure 3. The filament breakage time was longest for RC at 0.75 ± 0.02 s, which was significantly longer than 0.21 ± 0.03 s for TQ and 0.27 ± 0.01 s for TS (*p* < 0.05). Furthermore, a significant difference was observed between TQ and TS (*p* < 0.05). The dependence of the extensional viscosity on the Hencky strain is shown in Figure 4 and Table 2. Across all samples, the highest peak was observed between Hencky strain values of 3 and 4. For TQ and TS, the extensional viscosity decreased with increasing Hencky strain. Conversely, for RC, a decrease in extensional viscosity was noted with increasing Hencky strain; however, the extensional viscosity increased again when the Hencky strain exceeded 6. As shown in Table 2, the maximum extensional viscosity was higher for TQ compared with those for TS and RC; however, the difference was not significant. Notably, at a Hencky strain of 4, the extensional viscosity of TQ was significantly higher than that of TS, and at a Hencky strain of 5, the extensional viscosity of RC was significantly higher than that of TS. Furthermore, when the Hencky strain exceeded 6, the extensional viscosity of RC was significantly higher than those of both TQ and TS.

#### 3.1.3. Thixotropy

**Figure 5:**
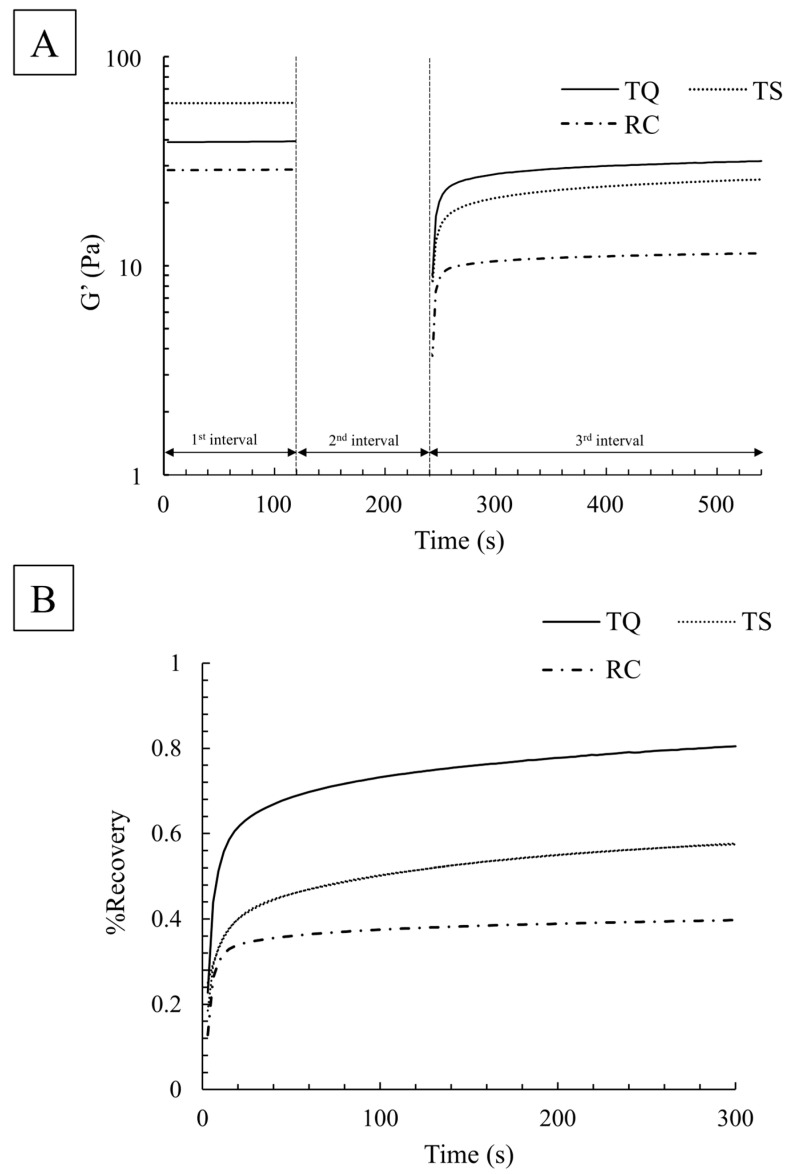
3ITT applied on TQ, TS, and RC. (**A**) Initial G′ (First interval) and time-dependent structural recovery (Third interval). (**B**) Time dependence of %Recovery after structural deformation.

The results of the 3ITT measurements are shown in Figure 5. In Figure 5A, a time-dependent recovery of the storage modulus G′ was observed after 240 s of structural deformation in the second interval, validating the thixotropic properties of all samples. The storage modulus G′ in the quiescent state before structural deformation in the first interval followed the order of TS > TQ > RC at all time points up to 120 s. However, during structural recovery in the third interval, the storage modulus G′ followed the order of TQ > TS > RC at all time points up to 540 s. The temporal transition of the recovery rate (% Recovery) after the structural deformation is shown in Figure 5B, with %Recovery following the order of TQ > TS > RC at all time points. The fitting parameters are listed in Table 3. The coefficient α_1_ of the large time constant k_1_, contributing to short-term structural recovery, was significantly higher for TQ compared with TS and RC (*p* < 0.05). Furthermore, the coefficient α_2_ of the small time constant k_2_, contributing to long-term structural recovery, was significantly lower for RC compared with TQ and TS (*p* < 0.05).

### 3.2. Swallowing Dynamics

#### 3.2.1. Muscle Activities

**Figure 6:**
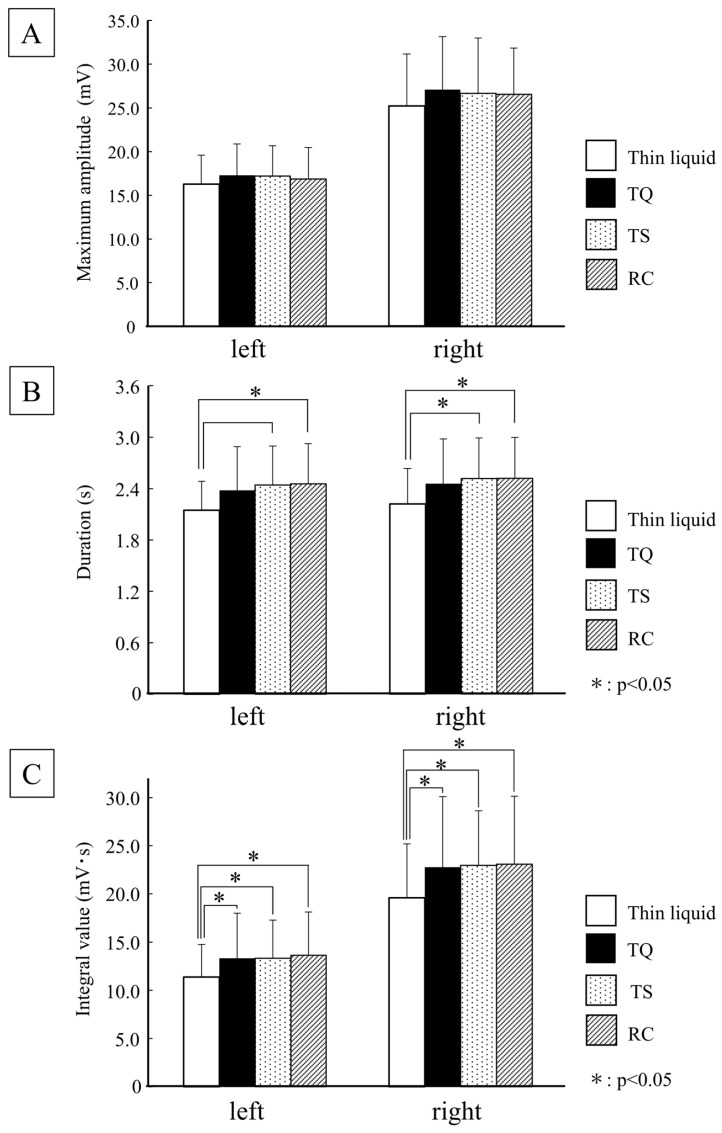
Suprahyoid muscle activities. (**A**) Maximum amplitude; (**B**) Duration; (**C**) Integral value.

The results of the maximum amplitude, duration, and integral value of the suprahyoid muscle activity on both sides during swallowing are shown in Figure 6. No difference was observed in the maximum amplitudes of the samples. The duration of suprahyoid muscle activity was significantly longer for TS and RC than for the thin liquid on both sides (*p* < 0.05). The integral value of the suprahyoid muscle activity was significantly larger for all samples of thickened liquids than for the thin liquid on both sides (*p* < 0.05).

#### 3.2.2. Tongue Pressure

**Figure 7:**
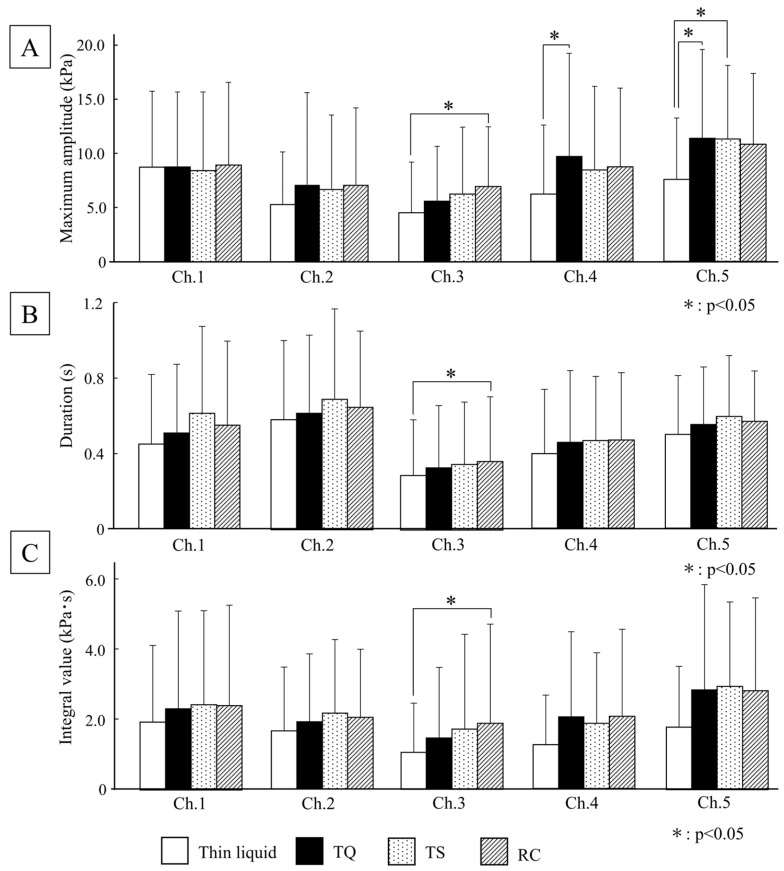
Tongue pressure. (**A**) Maximum amplitude; (**B**) Duration; (**C**) Integral value.

The results for the maximum tongue pressure, duration, and integral value are shown in Figure 7. Only RC demonstrated significantly higher values for maximum tongue pressure, duration, and integral value at the posterior-median region (Ch.3) compared with the thin liquid (*p* < 0.05). In addition, the maximum tongue pressure values in the right posterior circumferential region (Ch.4) of the TS and both posterior circumferential regions (Ch.4 and Ch.5) of the TQ were significantly higher than those for the thin liquid (*p* < 0.05).

#### 3.2.3. Swallowing Sound

**Figure 8:**
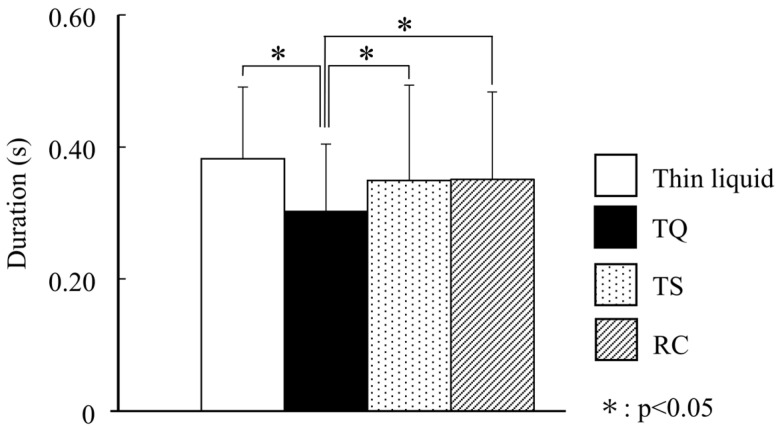
Swallowing sound.

The swallowing sound results are shown in Figure 8. The duration of the swallowing sound of TQ was significantly shorter compared with those of the thin liquid, TS, and RC (*p* < 0.05).

## 4. Discussion

To the best of our knowledge, this study represents the first attempt to investigate the variance in rheological properties among three commercial xanthan gum-based thickeners and their effects on swallowing dynamics. The research revealed variations in shear-thinning, extensional rheology, and thixotropy of the thickened liquids of each product, as well as differences in muscle activity, tongue pressure, and swallowing sounds produced by those liquids.

### 4.1. Methodological Consideration

Xanthan gum-based thickeners, commonly utilized for managing dysphagia, were specifically chosen for this study. The viscosity of the samples was standardized to a steady shear viscosity of 400 mPa∙s at a shear rate of 50 s^−1^. This viscosity level aligned with “Honey-like” in the U.S. National Dysphagia Diet, “Moderately Thick” in the Australian standards, and “Extremely Thick” in the Japanese Society for Dysphagia Rehabilitation [7,8]. Furthermore, the volume per swallow was 15 mL, similar to the standard intake volume for healthy individuals when swallowing water [24,25]. The effectiveness of this evaluation method was validated by revealing distinctions in swallowing dynamics among products through physiological measurements conducted at a clinically relevant viscosity level and a standard intake amount for healthy individuals. A previous study [26] comparing the pharyngeal transit time of a thickened bolus highlighted the crucial role of tongue pressure in facilitating safe bolus flow through the pharynx during swallowing. The reliability of the evaluation method used in this study, which measures tongue pressure during the oral propulsion phase, is deemed to be high. According to the report by Bolivar et al. [27], xanthan gum-based thickeners exhibit minimal viscosity changes within the temperature range of 20–40 °C and show high temporal stability of viscosity when prepared using clinically practical dissolution methods. Therefore, the influence of temperature increases resulting from contact with the oral or pharyngeal cavity during swallowing, as well as time-dependent changes in rheological properties after sample preparation, was considered to be minimal.

### 4.2. Shear and Extensional Rheology

In line with previous findings [10], this study confirmed that TQ, TS, and RC exhibit typical shear-thinning properties characteristic of xanthan gum-based thickeners. Notably, TQ demonstrated greater shear-thinning behavior compared with TS and RC, indicating its ease of flow owing to its low viscosity at around 300 s^−1^, a typical shear rate during the pharyngeal phase.

Furthermore, variations in extensional properties were observed among TQ, TS, and RC. RC displayed a longer filament breakage time, suggesting a spinnability characteristic. These distinctions may be attributed to the presence of coexisting substances, such as maltodextrin, potassium chloride, and organic acids in each xanthan gum-based thickener, as reported by Kongjaroen et al. [3].

In terms of thixotropy, differences in the recovery rates of the three products were observed. TQ exhibited significant and rapid recovery, whereas TS showed moderate recovery over time. Conversely, RC displayed minimal recovery in both short and long durations. Thixotropy is evaluated by the time to the point at which the network structure breaks down under external forces, and the time required to recover the original stable network structure when the external forces are removed [20]. This thixotropy is attributed to the electrical repulsion between the xanthan gum molecules [13]. The swift recovery of TQ compared with TS and RC can be attributed to the presence of electrolytes, such as trisodium citrate and calcium lactate, exclusively present in TQ, and potassium chloride solely in RC. These electrolytes likely affect the electrostatic repulsion between xanthan gum molecules.

### 4.3. Swallowing Dynamics

This study revealed that the integrated value of the suprahyoid muscle activity increased when compared with thin liquid across all samples. This result is consistent with that of previous research [28], which has shown that muscle activity increases with viscosity, suggesting a consistent effect associated with viscosity levels.

Furthermore, in the tongue pressure measurement, only RC demonstrated an increase in the maximum values, duration, and integrated value in the posterior-median region (Ch.3) when compared with thin liquid. This suggests that RC triggered tongue movements that exerted strong and long-term pressure on the posterior-median part of the palate during swallowing. Similarly, in TQ, the maximum tongue pressure on both the posterior circumferential regions (Ch.4 and Ch.5) was high, indicating that the tongue wrapped the bolus on both sides and propelled it toward the pharynx during swallowing.

Nakauma et al. [16] conducted a study on swallowing sounds and reported that thickened liquid with xanthan gum reduced flow velocity in the pharynx while shortening the duration of swallowing sounds by forming a cohesive bolus. In this study, the duration of swallowing sounds was longest for the thin liquid, likely due to the bolus being dispersed upon swallowing. Conversely, the duration of swallowing sounds was significantly shorter in TQ, suggesting that TQ formed the most cohesive bolus as it passed through the pharynx.

### 4.4. Rheological Characteristics and Swallowing Dynamics

This study suggests a potential relationship between rheological characteristics and swallowing dynamics during both oral transport and pharyngeal transit. Shear and extensional deformations were applied to the bolus during oral transport. The shear rate during oral transport was 50 s^−1^ [10], with no significant difference in viscosity observed between TQ, TS, and RC at this shear rate. However, RC exhibited spinnability, characterized by a prolonged filament breakage time. Tongue pressure measurements revealed that RC induced a forceful and long-term tongue movement pressing against the posterior-median region of the hard palate during swallowing (Ch.3). According to previous reports by Hori et al. [15], tongue pressure expression in the posterior-median region (Ch.3) aligns with the moment when the tip or end of the bolus passes through the upper esophageal sphincter or the isthmus of the mouth. Based on this information, the tongue probably exerts strong and long-term pressure on the posterior-median part (Ch.3) to propel the elongated RC bolus, resembling drawn threads, into the pharynx. Research on the sensory characteristics of thickened liquids [17] revealed a strong correlation between extensional viscosity at a Hencky strain of 8 and sensory spinnability. Consistent with these findings, RC showed higher extensional viscosity at a Hencky strain of 8 than TQ and TS. Our hypothesis, based on tongue pressure measurements, aligns with the sensory evaluation results. The study [17] also demonstrated a correlation between sensory cohesiveness and extensional viscosity within the Hencky strain range of 3–6. In this study, we found that the extensional viscosities of Hencky Strains 4 and 5 were significantly lower in TS than in TQ and RC. However, no specific physiological movements in the suprahyoid muscle activity or tongue pressure were solely influenced by TS. The lower Hencky strain region is thought to correspond to the timing of the initiation of bolus extension, which is the timing of the initiation of bolus propulsion. At that point, TS with low extensional viscosity may have impacted the coordination of Ch.1–5 to maintain the spreading bolus together because of its low extensional viscosity. Further research into the timing or order of Ch.1–5 may provide valuable insights.

During the pharyngeal phase, the bolus flows at a higher shear rate of 300 s^−1^, higher than that of oral transport [10]. The shear-thinning property of TQ was higher than those of TS and RC, and TQ demonstrated a significantly lower viscosity at 300 s^−1^, potentially leading to a shorter duration of swallowing sounds. Thixotropy may also have influenced the shorter duration of swallowing sounds. Based on the results of Matsubara et al. [29], the bolus swiftly traverses the pharynx, experiencing a momentary surge in pressure. The ability of TQ to rapidly regenerate its structure following breakdown in the pharynx may have influenced the observed shorter pharyngeal transit time.

Numerous rheological properties of thickened liquids impact the oral or pharyngeal phase, as indicated by Newman et al. [1]. Challenges in assessing these properties persist owing to limited information on factors such as molecular weight, chemical modification structure, and other characteristics of xanthan gum in commercially available xanthan gum-based thickeners. It may also be necessary to consider that the addition of maltodextrin during manufacture, as well as the granulation process itself, can influence the rheological properties of the product after dissolution [30]. While fundamental research in this area is important, it should be noted that thickeners are already being prescribed for patients with dysphagia. Our study, which examines the relationship between rheological properties and swallowing dynamics in xanthan gum-based thickeners, therefore provides practical, clinically relevant insights. This study did not assess participants’ perceptions or palatability of the thickened liquids. Considering previous report demonstrating strong correlations between the rheological properties of thickened fluids and their sensory characteristics [16], it is possible that the perceived texture and palatability influenced the physiological movements.

The study participants were young and healthy, with no swallowing disorders. A previous study by Hadde et al. [26] examined the impact of different thickened liquids on swallowing dynamics using videofluoroscopy. The study highlighted the high safety of swallowing in healthy individuals and emphasized the need for further research on individuals with dysphagia. Differences in tongue pressure strength, coordinated tongue movements, and pharyngeal pressure have been reported in older adults or individuals with dysphagia compared with young healthy adults [31,32,33], suggesting that bolus propulsion in the oral and pharyngeal phases may differ from that of healthy individuals. Therefore, a clinical trial should be conducted to assess the rheological properties of xanthan gum-based thickeners on swallowing dynamics during the oral and pharyngeal phases in patients with dysphagia, to enhance our understanding of safe swallowing practices. In this study, distilled water was selected to eliminate the influence of the solvent on viscosity. However, considering previous reports, such as Kim et al. [34], which examined the effects of thickening on the rheological properties, flavor, and texture of juices and nutrient-dense beverages, we aim to include several dispersing media. Despite these limitations, this study is the first to elucidate variations in shear-thinning, extensional properties, and thixotropy among xanthan gum-based thickeners commonly used in clinical settings. Additionally, the study involved healthy individuals to examine differences in suprahyoid muscle activity, tongue pressure, and swallowing sounds among these products. Of particular value is the clarification of the relationship between rheological properties and bolus flow, as well as deformation through tongue pressure measurements. The findings of this study are expected to significantly contribute to the development and enhancement of new thickeners, as well as the selection of xanthan gum-based thickeners in clinical practice. Further study is needed to evaluate the rheological properties of xanthan gum-based thickeners on swallowing dynamics in dysphagia patients, to enhance understanding of safe swallowing practices. Finally, although the present study focused on liquid boluses for dehydration, innovations in texture-modified diets (TMDs)—such as pureed diets and fork-mashable diets—are expected to reduce the risk of malnutrition in patients with dysphagia [35]. Our findings may contribute to elucidating the relationship between the food characteristics of TMDs, such as hardness, adhesiveness, cohesiveness, and the physiological movements during swallowing.

## 5. Conclusions

This study revealed the variations in shear-thinning, extensional properties, and thixotropy among xanthan gum-based thickeners. We measured suprahyoid muscle activity, tongue pressure, and swallowing sounds to assess the influence of these physical property differences on swallowing dynamics in young healthy individuals. Our findings indicate that the extensional properties of thickened liquid could potentially impact tongue pressure during the propulsion to the pharynx, whereas shear-thinning and thixotropy may influence the duration of swallowing sounds.

## Figures and Tables

**Table 1:** Power-law parameters and apparent shear viscosity at 40 s^−1^ and 251 s^−1^.

Sample	Power-Law Parameters		Apparent Shear Viscosity (mPa∙s)	
	K	*n*	40 s^−1^	251 s^−1^
TQ	11.7 ± 0.50 ^a^	0.142 ± 0.001 ^a^	485 ± 22 ^a^	99.2 ± 4.4 ^a^
TS	10.1 ± 0.17 ^b^	0.163 ± 0.001 ^b^	512 ± 1 ^a^	122.1 ± 1.4 ^b^
RC	9.9 ± 0.25 ^b^	0.165 ± 0.002 ^b^	508 ± 7 ^a^	127.6 ± 2.2 ^b^

Note: Data are presented as means ± standard deviations of triplicates. Data with different superscripts in the same column differ significantly (*p* < 0.05).

**Table 2:** Maximum extensional viscosity and extensional viscosity at each Hencky strain for TQ, TS, and RC.

Sample	Maximum Extensional Viscosity (Pa∙s)	Extensional Viscosity at Each Hencky Strain (Pa∙s)					
		3	4	5	6	7	8
TQ	76.1 ± 17.9 ^a^	24.2 ± 8.7 ^a^	24.9 ± 3.0 ^a^	8.2 ± 0.8 ^ab^	5.6 ± 0.0 ^a^	4.6 ± 0.2 ^a^	4.1 ± 0.2 ^a^
TS	42.7 ± 9.6 ^a^	14.9 ± 10.3 ^a^	6.5 ± 0.9 ^b^	5.1 ± 0.9 ^a^	4.0 ± 0.3 ^a^	3.6 ± 0.3 ^a^	3.8 ± 0.5 ^a^
RC	45.5 ± 1.9 ^a^	35.8 ± 9.9 ^a^	19.7 ± 7.4 ^ab^	12.9 ± 2.8 ^b^	10.0 ± 1.6 ^b^	11.3 ± 2.9 ^b^	12.5 ± 3.5 ^b^

Note: Data are presented as means ± standard deviations of triplicates. Data with different superscripts in the same column differ significantly (*p* < 0.05).

**Table 3:** Fitting parameters of Time-dependence of %Recovery values on structural regeneration.

Sample	Parameters				
	α_1_	k_1_	α_2_	k_2_	β
TQ	0.394 ± 0.006 ^a^	0.128 ± 0.003 ^a^	0.202 ± 0.006 ^a^	8.2 ± 0.5 × 10^−3 a^	0.223 ± 0.008 ^a^
TS	0.206 ± 0.028 ^b^	0.097 ± 0.010 ^b^	0.195 ± 0.020 ^a^	6.9 ± 0.4 × 10^−3 b^	0.198 ± 0.018 ^a^
RC	0.238 ± 0.007 ^b^	0.188 ± 0.009 ^c^	0.064 ± 0.002 ^b^	9.2 ± 0.3 × 10^−3 a^	0.098 ± 0.009 ^b^

Note: Data are presented as means ± standard deviations of triplicates. Data with different superscripts in the same column differ significantly (*p* < 0.05).

## Data Availability

The data supporting the findings of this study are available from the corresponding author upon reasonable request.

## Abbreviations

The following abbreviations are used in this manuscript:

EMG	Electromyography
RC	Resource Thicken Up Clear
TQ	Tsururinko Quickly
TS	Toromi Smile
TMDs	Texture-modified diets
